# Stray Cat Colonies Lacking Health Surveillance and Management Pose Infection Pressure for *Aelurostrongylus abstrusus* on Sympatric Domestic and Wild Felids

**DOI:** 10.3390/ani14233400

**Published:** 2024-11-25

**Authors:** Diana Gassó, Jorge Ramón López-Olvera, Gregorio Mentaberre

**Affiliations:** 1 Wildlife Ecology and Health Group (WE&H), Departament de Ciència Animal, Escola Tècnica Superior d’Enginyeria Agroalimentària i Forestal i de Veterinària (ETSEAFiV), Universitat de Lleida (UdL), 25199 Lleida, Spain; gregorio.mentaberre@udl.cat; 2 WE&H, Servei d’Ecopatologia de Fauna Salvatge (SEFaS), Departament de Medicina i Cirurgia Animals, Universitat Autònoma de Barcelona, 08193 Bellaterra, Spain; elrebeco@yahoo.es

**Keywords:** *Aelurostrongylus abstrusus*, street colony cats, lung nematodes, shelter cats, Spain

## Abstract

The prevalence of respiratory nematodes in domestic animals has increased in Europe in recent decades. *Aelurostrongylus abstrusus* is the most common parasitic nematode of the respiratory tract in felids, and an increase in its prevalence has been suggested in different European countries including Spain, with values ranging from 0% to 30%. The Baermann technique was used to detect metastrongyloid larvae in 93 faecal samples from cats living in colonies (n = 29), cats living in shelters (n = 30), and owned cats (n = 34). Eleven cats (11.2%) were infected by *A. abstrusus*, the only species detected. The prevalence in colony cats (34.5%, 10/29) was significantly higher than the 3.3% (1/30) prevalence in shelter cats. Conversely, *A. abstrusus* was not found in any of the owned cats. The results can be representative of other areas in the Mediterranean basin, indicating the need to improve feline management and knowledge about the distribution and abundance of this parasite through geographically wider studies in Spain. This study emphasizes the importance of deworming domestic cats, especially those from street and rural environments or with outdoor lifestyles. The results also urge the need for population and health control of street cat colonies. Incorporating a deworming protocol in the current trapping–neutering–release campaigns for cat population control will be essential.

## Simple Summary

This study investigated the prevalence of lungworm infections in cats from different environments in Catalonia, Spain. A total of 93 cats from street colonies, shelters, and owned households were tested for lung nematodes. *Aelurostrongylus abstrusus* was detected in 34.5% of the cats from street colonies, as well as in one shelter cat, but not in any of the owned cats. These findings suggest that uncontrolled stray cat colonies pose a significant health risk to owned and wild felids due to the high prevalence of lungworm infections. The study emphasizes the need for managing cat populations, particularly through deworming and health protocols, to reduce the risk of infection. Including such measures in cat colony management programmes, like capture–sterilization–-release campaigns, is crucial to controlling the spread of lungworm.

## 1. Introduction

In recent decades, the observed prevalence and distribution of cat respiratory nematodes in Europe have increased [1], with first-time detections of these parasites in countries such as Albania [2] and Sweden [3]. For years, the prevalence of lungworms described in different European countries including Spain ranged between 1% and 2% [4,5,6,7,8], although higher prevalences around 10% had been reported in other European countries such as Portugal [9], Italy [10], and Germany [11]. In Italy, cat lungworm parasites are endemic, with prevalences reaching up to 38% [12,13]. However, higher prevalences in cat faeces ranging from 6.5% to 55.2% have been more recently reported in different regions within Spain [1,14,15]. These numbers increase to values between 20% and 30% when considering seroprevalence in cats [16,17,18], because false negative coproscopic results occur during prepatent infections and due to the irregularity of larval shedding [19]. Lungworms have also been reported in Asia, America, and Australia [20]. Globalization and global warming have been pointed to as principal factors favouring the spread of lungworms [21], which require an intermediate host to complete their life cycle. Both the parasites and their intermediate hosts (slug or snail) can adapt to climate change [22], facilitating their dispersion and the increase in their distribution range. However, few studies have been conducted on the intermediate [23] and even less on the paratenic [24] hosts, highlighting the need for further investigation in this area.

These widely distributed parasites infect domestic cats (*Felis catus* Linnaeus 1758) [25,26,27] but also their wild counterparts, European wildcats (*Felis silvestris* Schreber 1777) and other wild felids [28,29]. The clinical signs caused by lungworms range from asymptomatic to severe bronchopneumonia and death [30,31,32,33].

The lungworm *Aelurostrongylus abstrusus* Railliet 1898 (Nematode: Rhabditida: Angiostrongylidae) is the most common parasitic nematode of the respiratory tract in felids, which are mainly infected by eating infected small rodents that act as paratenic hosts. *Aelurostrongylus abstrusus* is found throughout the world, with prevalences reported in Europe ranging from 0.4 to 43.1% [5]. Other lungworms, such as *Troglostrongylus* spp. (Crenosomatidae) and *Oslerus rostratus* Gerichter 1945 (Filaroididae), were neglected in the past due to similarities in their first-stage larvae (L1), which are used for diagnosis, with the more abundant *A. abstrusus*. Although they have been more frequently identified in recent years thanks to the better characterization of their morphological differences and the molecular data availability [34,35], they continue to be detected in low numbers in domestic cats [36], in wildlife [28,29], or in cats living in sympatry with wild felids [12,18,37,38]. However, *Troglostrongylus brevior* Gerichter 1948 has specifically been increasingly reported in domestic cats in Europe [1,28,36,39]. In European felids, other lung nematodes are also present, including *Capillaria aerophila* Creplin 1839 (Trichurida: Capillariidae) [36], which is distinguishable by its characteristic lemon-shaped eggs that are excreted in faeces. Additionally, the cardiopulmonary nematodes *Angiostrongylus chabaudi* Biocca 1957 (Angiostrongylidae) [40] and *Metathelazia massinoi* Davtian 1933 (Spirurida: Pneumospiruridae)—also known as *Vogeloides massinoi* and *Osleroides massino* [41,42]—although less commonly observed, have also been described in wild hosts.

While cats’ lifestyle, mainly outdoor access, is acknowledged as a hazard factor for lungworm infection [1,14,28,36,43], few studies have compared the prevalence of lungworms between shelter cats and street-colony-dwelling cats in Europe [44]. These prevalences are likely to differ, because street-colony-dwelling cats do not receive any treatment, while shelter cats receive effective treatments to control lungworms [1,20,45,46]. However, no studies in Spain have compared lungworm prevalences based on cats’ lifestyle.

There is therefore a lack of knowledge regarding the reasons for the variability and the apparent recent increase in the prevalence of lungworms in domestic cats in Europe overall and specifically in Spain, as well as the effect of cats’ lifestyle and outdoor access on such a prevalence. The objective of this study is to assess the prevalence of cat lung metastrongyloids and the effect of cats’ lifestyle in a specific geographic area of Catalonia, northeastern Spain.

## 2. Materials and Methods

### 2.1. Study Area

**Figure 1:**
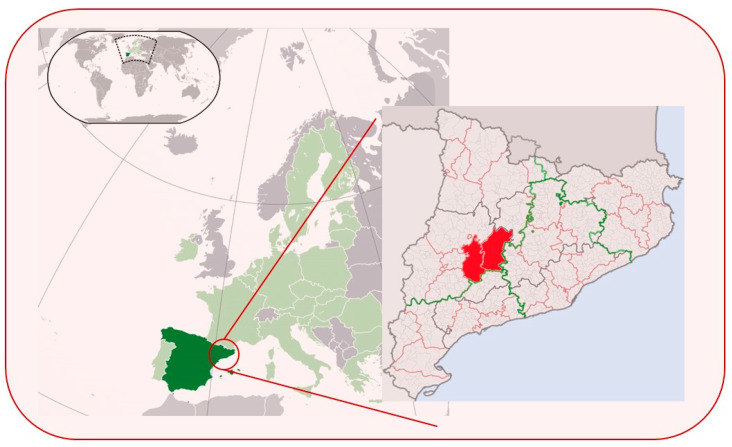
Map of the study area. Spain is marked in dark green. Catalonia region is shown in the inset, with the study areas of Urgell and Segarra indicated in red.

The province of Lleida, situated in northeastern Spain within Catalonia, boasts diverse landscapes ranging from the Pyrenees to the Ebro River basin. Characterized by agricultural plains, valleys, and mountainous areas, it presents a unique gradient of anthropization and climate. Our study areas, primarily in the Urgell and Segarra, encompass diverse rural environments with the potential for domestic–wildlife interactions (Figure 1). Previous studies found that interactions between domestic and wild carnivores may be most likely to occur in rural locations [47,48].

### 2.2. Faecal Samples and Data Collection

From April 2023 to Jun 2024, 93 faecal samples were collected from cat colonies (n = 29), cat shelters (n = 30), and owned cats (n = 34, including eight with outdoor access and 26 always kept indoors, Table 1). Fresh faecal samples were directly collected from outdoor sand, trap boxes, or commercial cat litter, respectively. All the faecal samples were subjected to a modified Baermann technique [49]. The Baermann test was performed using 5 to 10 g of faeces wrapped in a double layer of gauze and placed in a sedimentation cup filled with water at environmental temperature. After 12–24 h, the supernatant was discarded, and the sediment (10 mL) was analysed with a stereoscopic magnifying glass. When a total larvae count was impossible due to their abundance, a McMaster chamber was used for quantification. Metastrongyloid larvae L1 of *A. abstrusus* were identified by key morphological and morphometric features, particularly their total length and tail and head shape [25,35,45,46]. The differential features of metastrongyloid larvae found in feline faecal samples have been previously described [25].

The characteristics of the cats included in this study are presented in Table 1.

### 2.3. Data Analysis

The descriptive analysis of the data and a two-proportion z-test were performed using R software version 4.4.0 (R Core Team, 2024 [50]).

## 3. Results

**Figure 2:**
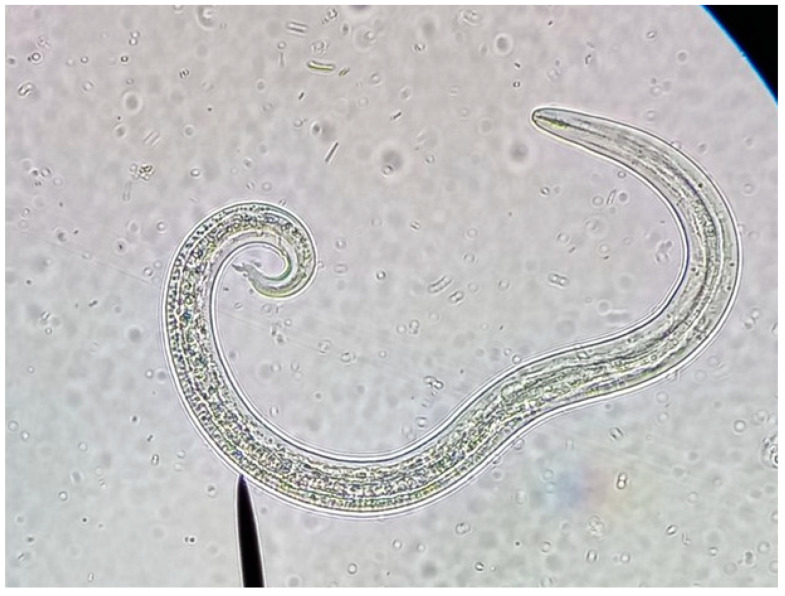
First-stage larva of *Aelurostrongylus abstrusus*, with the anterior end showing a rounded extremity and a terminal oral opening and a kinked tail with distinct knob-like or small finger-like projections at the tip of the cuticular spines.

Of the 93 cats, 11 (11.8%, CI95 5.3–19.4) were infected by *A. abstrusus* (Figure 2). Ten of the infested cats were from colonies (10/29, 34.5%, CI95 17.2–51.8) and one was from a shelter (1/30, 3.3%, CI95 0.0–9.8), resulting in a statistically significant higher prevalence in street cats than in shelter cats (X-squared = 7.4909; df = 1; *p*-value = 0.006201). The parasite was not detected in any of the owned cats (0/34).

The mean intensity was 1365 larvae (min–max: 2–12,000; sd: 13,988.2), and the only shelter cat who was infected had an intensity of 36 larvae, all found in ten grammes of faeces.

## 4. Discussion

The prevalence of the lungworm *A. abstrusus* observed in this study was overall similar to those found in previous and recent studies conducted in Spain [1,14,15]. However, the specific prevalence in the street-colony-dwelling cats was higher, similar to previous reports in Bulgaria (35.8%) [1], and even higher than the 25% seroprevalence described in endemic areas of Italy [17]. Since coprological techniques are less sensitive than serology [19], the real exposure to *A. abstrusus* of the cats sampled in this study could be even higher. This tendency of a higher prevalence in stray cats compared to shelter cats and, finally, owned ones, has been previously reported for intestinal parasites and lungworms, including *A. abstrusus* [10,44]. The higher prevalence of *A. abstrusus* in street colony cats found in this study matches with the high metastrongyloid infection rates reported in outdoor cats in the Canary Islands [14] and agrees with previous studies from Italy and Switzerland in identifying an outdoor lifestyle as a risk factor, supported by prevalence data for lungworms, including *A. abstrusus* [10,51]. However, to our knowledge, this is one of the first studies to examine the relationship between *A. abstrusus* prevalence and cats’ lifestyle specifically in the Mediterranean basin, which is particularly relevant. Given the current climate change conditions in the region, future increases in the range of helminth infections toward northern latitudes in Europe can be anticipated [21,52].

Outdoor access has already been identified as a risk factor for lungworm and specifically *A. abstrusus* infection in domestic cats [5,10,44,51,53]. This higher outdoor hazard could be related to the infection pressure posed by wild carnivores, who have a higher lungworm prevalence than domestic ones [28,29,54,55,56] and could serve as a reservoir for *T. brevior* and *Eucoleus aerophilus* (syn. *Capillaria aerophila*), leading to high prevalence and co-infections where domestic and wild cats coexist [37,57]. In our study area, *A. abstrusus* infections may similarly be maintained through reservoir hosts. Here, street colony domestic cats, who generally lack the deworming practises applied to home-kept cats, may play a similar role to wild reservoirs by posing an infection risk to domestic cats. The presence of European wildcat in the region [58] further supports the potential for cross-species infection pressure. Further studies including this wild reservoir of lung nematodes in general and *A. abstrusus* specifically are required to investigate the role of this species in the shared epidemiology of these parasites from a One Health approach [55,59].

The lack of finding of lungworm species other than *A. abstrusus* agrees with recent reports of this species as the most important respiratory nematode in cats [57], although *T. brevior* and *E. aerophilus* are becoming increasingly relevant in feline clinical parasitology [45]. Similar results, with a single infecting species, have been observed in northeastern Spain [15] and other countries like Ireland [6]. Although previous issues with misdiagnosis in lungworm species have been resolved [57,60], the use of molecular techniques such as PCR would be preferable [18,34], not only for species identification but also to explore the epidemiological cycles among host species and populations.

While this study contributes new data on the *A. abstrusus* prevalence in domestic cats according to their management condition in the Mediterranean basin, prevalences can vary widely locally [61]. More comprehensive studies involving larger sample sizes from diverse geographic areas are necessary to achieve a more accurate and complete understanding of the epidemiology of *A. abstrusus* in Mediterranean environments. Moreover, our results may be conservative, as the prevalence could be higher due to the limitations and low sensitivity of coprological tests [13,19]. Although serological studies generally yield higher prevalence estimates [11,16,17,51] by detecting prepatent individuals, they can also produce higher prevalence rates due to false positives from cross-reactions with other metastrongyloid parasites or false negatives in immunosuppressed cats [19]. Therefore, coprological and serological methods should be combined to obtain more reliable results.

The impact of domestic cats with outdoor access and stray and feral cats on biodiversity has been repeatedly reported and demonstrated [59,62,63]. Besides predation, domestic cats have also been responsible for causing a feline leukaemia virus epidemic with mortality in the endangered Iberian lynx (*Lynx pardinus* Temminck 1982) population in Doñana, southwestern Spain [64]. While the prevalences of *A. abstrusus* and other lungworms in wild felids are among the highest values reported [55,56], their contributions to environmental burdens and infection pressures depend not only on the individual burden but mainly on the shedding rate and abundance of each population and species [65]. Untreated and uncontrolled street colony cats could act epidemiologically as highly prevalent and highly infected overabundant hosts, individually equalling wild felids but demographically overcoming their role due to their anthropically subsidized populations [62,63,66,67,68]. Therefore, monitoring not only the prevalence and parasitic load, but also the shedding rate and population density and/or abundance of both domestic and wild hosts [62] is essential to understanding the complexity of the epidemiological scenario of *A. abstrusus* and other lungworms in the interface between domestic and wild felids.

To understand domestic cat population dynamics, the collective population of owned cats, unowned (including feral) cats, and cats in the shelter system must be considered simultaneously, since each subpopulation contributes differently to the overall cat population within a community [66]. Such population monitoring and knowledge should also include the sympatric wild felid populations, which is currently European wildcat in our study area. The “One Health One Welfare” approach, which encompasses human, domestic, and wild animals, as well as ecosystem health, also requires an understanding of the population dynamics and disease epidemiology in each one of these host groups [59].

Our study adds a new population health hazard to the already known biodiversity conservation threats posed by street cat colonies [64,68], highlighting the need and urgency of their population and health monitoring. However, the current management of feral cats and stray cat colonies relies mostly on the trap–neutering–release (TNR) system [69], which has repeatedly been demonstrated to be ineffective [70]. Nevertheless, if continued despite their lack of success, our results support the recommendation that such TNR campaigns incorporate a deworming protocol to treat cats, and potentially a vaccination protocol for contagious viral diseases, such as feline leukaemia virus, to reduce the risk of sympatric threatened wildlife felids [64]. These additional measures will not only protect the welfare of unowned cats but also reduce health risks to owned cats, wildlife, and, ultimately, human health. In particular, deworming can help reduce the prevalence of *Toxocara* spp. and other intestinal parasites, decreasing the risk of zoonotic infections such as larva *migrans* [71].

Veterinary practitioners from Catalonia should consider lung nematodes in the differential diagnosis of cats with respiratory signs, particularly those from street colonies, shelters, or with outdoor access and not properly dewormed. Conducting a Baermann test (an easy and economical method) is essential to avoid misdiagnosing lungworms as asthma or other pulmonary conditions [72].

## 5. Conclusions

The prevalence of *A. abstrusus* was consistent with both previous and recent studies conducted in Spain; however, the prevalence among colony-dwelling street cats was significantly higher, acting as an uncontrolled potential source of infection. This study adds lungworms, specifically *A. abstrusus,* to the known biodiversity and health risks posed by stray domestic cat colonies.

## Figures and Tables

**Table 1:** Features of the cats sampled in this study.

		Cat Colonies	Cat Shelter	Owned Cats	Total
Sample size		29	30	34	93
Known age (years)		5	1	28	35
	Mean	0.85	4.5	3.58	3.4
	SD	0.34	NA	2.31	2.56
	Minimum	0.25	NA	0.1	0.1
	Maximum	10	NA	6	10
Known sex		10	3	34	47
	Female	4	3	17	24 (51%)
	Male	6	0	17	23 (49%)
Endoparasitic treatment *					
Yes		0	30	21	51 (61.4%)
	<6 months ago		30	11	41 (49.4%)
	>6 months ago		0	9	9 (10.8%)
	Unknown			1	1 (1.1%)
No		29	0	3	32 (38.4%)

SD = Standard deviation; * milbemycin oxime/praziquantel at manufacturer-recommended dosages.

## Data Availability

The datasets supporting the conclusions of this article are included within the article.

## References

[1] Giannelli A., Capelli G., Joachim A., Hinney B., Losson B., Kirkova Z., René-Martellet M., Papadopoulos E., Farkas R., Napoli E. (2017). Lungworms and Gastrointestinal Parasites of Domestic Cats: A European Perspective. Int. J. Parasitol..

[2] Knaus M., Kusi I., Rapti D., Xhaxhiu D., Winter R., Visser M., Rehbein S. (2011). Endoparasites of Cats from the Tirana Area and the First Report on *Aelurostrongylus abstrusus* (Railliet, 1898) in Albania. Wien. Klin. Wochenschr..

[3] Grandi G., Comin A., Ibrahim O., Schaper R., Forshell U., Lind E.O. (2017). Prevalence of Helminth and Coccidian Parasites in Swedish Outdoor Cats and the First Report of *Aelurostrongylus abstrusus* in Sweden: A Coprological Investigation. Acta Vet. Scand..

[4] Bourgoin G., Callait-Cardinal M.-P., Bouhsira E., Polack B., Bourdeau P., Roussel Ariza C., Carassou L., Lienard E., Drake J. (2022). Prevalence of Major Digestive and Respiratory Helminths in Dogs and Cats in France: Results of a Multicenter Study. Parasites Vectors.

[5] Elsheikha H.M., Wright I., Wang B., Schaper R. (2019). Prevalence of Feline Lungworm *Aelurostrongylus abstrusus* in England. Vet. Parasitol. Reg. Stud. Rep..

[6] Garcia-Campos A., Power C., O’Shaughnessy J., Browne C., Lawlor A., McCarthy G., O’Neill E.J., De Waal T. (2019). One-Year Parasitological Screening of Stray Dogs and Cats in County Dublin, Ireland. Parasitology.

[7] Henry P., Huck-Gendre C., Franc M., Williams T.L., Bouhsira E., Lienard E. (2022). Epidemiological Survey on Gastrointestinal and Pulmonary Parasites in Cats around Toulouse (France). Helminthologia.

[8] Miró G., Montoya A., Jiménez S., Frisuelos C., Mateo M., Fuentes I. (2004). Prevalence of Antibodies to *Toxoplasma gondii* and Intestinal Parasites in Stray, Farm and Household Cats in Spain. Vet. Parasitol..

[9] Nabais J., Alho A.M., Gomes L., Vicente G., de Carvalho L.M. (2014). *Aelurostrongylus abstrusus* in Cats and *Angiostrongylus vasorum* in Dogs from Lisbon, Portugal. Acta Parasitológica Port..

[10] Genchi M., Vismarra A., Zanet S., Morelli S., Galuppi R., Cringoli G., Lia R., Diaferia M., Frangipane Di Regalbono A., Venegoni G. (2021). Prevalence and Risk Factors Associated with Cat Parasites in Italy: A Multicenter Study. Parasites Vectors.

[11] Schnyder M., Schaper R., Gori F., Hafner C., Strube C. (2021). *Aelurostrongylus abstrusus* Antibody Seroprevalence Reveals that Cats are at Risk of Infection throughout Germany. Pathogens.

[12] Traversa D., Morelli S., Cassini R., Crisi P.E., Russi I., Grillotti E., Manzocchi S., Simonato G., Beraldo P., Viglietti A. (2019). Occurrence of Canine and Feline Extra-Intestinal Nematodes in Key Endemic Regions of Italy. Acta Trop..

[13] Vismarra A., Schnyder M., Strube C., Kramer L., Colombo L., Genchi M. (2023). Diagnostic Challenges for *Aelurostrongylus abstrusus* Infection in Cats from Endemic Areas in Italy. Parasites Vectors.

[14] García-Livia K., Reyes R., Amaro-Ramos V., Baz-González E., Martin-Carrillo N., Rodríguez-Ponce E., Foronda P. (2023). Metastrongyloid Infection with *Aelurostrongylus abstrusus*, *Troglostrongylus brevior*, *Oslerus rostratus* and *Angiostrongylus chabaudi* in Feral Cats from the Canary Islands (Spain). Animals.

[15] Remesar S., García-Dios D., Calabuig N., Prieto A., Díaz-Cao J.M., López-Lorenzo G., López C., Fernández G., Morrondo P., Panadero R. (2022). Cardiorespiratory Nematodes and Co-infections with Gastrointestinal Parasites in New Arrivals at Dog and Cat Shelters in North-western Spain. Transbound. Emerg. Dis..

[16] Cavalera M.A., Schnyder M., Gueldner E.K., Furlanello T., Iatta R., Brianti E., Strube C., Colella V., Otranto D. (2019). Serological Survey and Risk Factors of *Aelurostrongylus abstrusus* Infection among Owned Cats in Italy. Parasitol. Res..

[17] Di Cesare A., Gueldner E.K., Traversa D., Veronesi F., Morelli S., Crisi P.E., Pampurini F., Strube C., Schnyder M. (2019). Seroprevalence of Antibodies against the Cat Lungworm *Aelurostrongylus abstrusus* in Cats from Endemic Areas of Italy. Vet. Parasitol..

[18] Morelli S., Diakou A., Di Cesare A., Schnyder M., Colombo M., Strube C., Dimzas D., Latino R., Traversa D. (2020). Feline Lungworms in Greece: Copromicroscopic, Molecular and Serological Study. Parasitol. Res..

[19] Zottler E.-M., Strube C., Schnyder M. (2017). Detection of Specific Antibodies in Cats Infected with the Lung Nematode *Aelurostrongylus abstrusus*. Vet. Parasitol..

[20] Raue K., Rohdich N., Hauck D., Zschiesche E., Morelli S., Traversa D., Di Cesare A., Roepke R.K.A., Strube C. (2021). Efficacy of Bravecto^®^ Plus Spot-on Solution for Cats (280 Mg/Ml Fluralaner and 14 Mg/Ml Moxidectin) for the Prevention of Aelurostrongylosis in Experimentally Infected Cats. Parasites Vectors.

[21] Traversa D., Di Cesare A., Conboy G. (2010). Canine and Feline Cardiopulmonary Parasitic Nematodes in Europe: Emerging and Underestimated. Parasites Vectors.

[22] Traversa D., Di Cesare A. (2014). Cardio-Pulmonary Parasitic Nematodes Affecting Cats in Europe: Unraveling the Past, Depicting the Present, and Predicting the Future. Front. Vet. Sci..

[23] Segeritz L., Cardona A., Taubert A., Hermosilla C., Ruiz A. (2021). Autochthonous *Angiostrongylus cantonensis*, *Angiostrongylus vasorum* and *Aelurostrongylus abstrusus* Infections in Native Terrestrial Gastropods from the Macaronesian Archipelago of Spain. Parasitol. Res..

[24] Colella V., Knaus M., Lai O., Cantile C., Abramo F., Rehbein S., Otranto D. (2019). Mice as Paratenic Hosts of *Aelurostrongylus abstrusus*. Parasites Vectors.

[25] Traversa D., Di Cesare A. (2016). Diagnosis and Management of Lungworm Infections in Cats: Cornerstones, Dilemmas and New Avenues. J. Feline Med. Surg..

[26] Bowman D.D., Hendrix C.M., Lindsay D.S., Barr S.C. (2002). The Nematodes. Feline Clinical Parasitology.

[27] Conboy G. (2009). Helminth Parasites of the Canine and Feline Respiratory Tract. Vet. Clin. N. Am. Small Anim. Pract..

[28] Bisterfeld K., Raulf M.-K., Waindok P., Springer A., Lang J., Lierz M., Siebert U., Strube C. (2022). Cardio-Pulmonary Parasites of the European Wildcat (*Felis silvestris*) in Germany. Parasites Vectors.

[29] Deak G., Ionică A.M., Pop R.A., Mihalca A.D., Gherman C.M. (2022). New Insights into the Distribution of Cardio-Pulmonary Nematodes in Road-Killed Wild Felids from Romania. Parasites Vectors.

[30] Febo E., Crisi P.E., Traversa D., Luciani A., Di Tommaso M., Pantaleo S., Santori D., Di Cesare A., Boari A., Terragni R. (2019). Comparison of Clinical and Imaging Findings in Cats with Single and Mixed Lungworm Infection. J. Feline Med. Surg..

[31] Napoli E., Pugliese M., Basile A., Passantino A., Brianti E. (2023). Clinical, Radiological, and Echocardiographic Findings in Cats Infected by *Aelurostrongylus sbstrusus*. Pathogens.

[32] Giannelli A., Passantino G., Ramos R.A.N., Lo Presti G., Lia R.P., Brianti E., Dantas-Torres F., Papadopoulos E., Otranto D. (2014). Pathological and Histological Findings Associated with the Feline Lungworm *Troglostrongylus brevior*. Vet. Parasitol..

[33] Murad B., Yankova S., Shiron M., Tonev A., Iliev P., Kirkova Z., Tsachev I. (2019). Clinical Cases of *Aelurostrongylus abstrusus* and Feline Immunodeficiency Virus Co-infection in Cats. Tradit. Mod. Vet. Med..

[34] Di Cesare A., Veronesi F., Frangipane Di Regalbono A., Iorio R., Traversa D. (2015). Novel Molecular Assay for Simultaneous Identification of Neglected Lungworms and Heartworms Affecting Cats. J. Clin. Microbiol..

[35] Otranto D., Brianti E., Dantas-Torres F. (2013). *Troglostrongylus brevior* and a Nonexistent ‘Dilemma’. Trends Parasitol..

[36] Di Cesare A., Di Francesco G., Frangipane Di Regalbono A., Eleni C., De Liberato C., Marruchella G., Iorio R., Malatesta D., Romanucci M.R., Bongiovanni L. (2015). Retrospective Study on the Occurrence of the Feline Lungworms *Aelurostrongylus abstrusus* and *Troglostrongylus* Spp. in Endemic Areas of Italy. Vet. J..

[37] Dimzas D., Morelli S., Traversa D., Di Cesare A., Van Bourgonie Y.R., Breugelmans K., Backeljau T., Di Regalbono A.F., Diakou A. (2020). Intermediate Gastropod Hosts of Major Feline Cardiopulmonary Nematodes in an Area of Wildcat and Domestic Cat Sympatry in Greece. Parasites Vectors.

[38] Salant H., Yasur-Landau D., Rojas A., Otranto D., Mazuz M.L., Baneth G. (2020). *Troglostrongylus brevior* Is the Dominant Lungworm Infecting Feral Cats in Jerusalem. Parasitol. Res..

[39] Deak G., Ionică A.M., Mihalca A.D., Gherman C.M. (2017). *Troglostrongylus brevior*: A New Parasite for Romania. Parasites Vectors.

[40] Giannelli A., Kirkova Z., Abramo F., Latrofa M.S., Campbell B., Zizzo N., Cantacessi C., Dantas-Torres F., Otranto D. (2016). *Angiostrongylus chabaudi* in Felids: New Findings and a Review of the Literature. Vet. Parasitol..

[41] Panova O.A., Khrustalev A.V., Porfiryeva L.Y. (2022). Review of pulmonary nematodoses of domestic cats with description of the first case of aelurostrongylosis in a cat in Russia. Russ. J. Parasitol..

[42] Davtian E.A. (1933). Ein neuer Nematode aus den Lungen der Hauskatze. *Osleroides* massino, nov. sp. Deutsch. Tierarztl. Wochenschr..

[43] Mircean V., Titilincu A., Vasile C. (2010). Prevalence of Endoparasites in Household Cat (*Felis catus*) Populations from Transylvania (Romania) and Association with Risk Factors. Vet. Parasitol..

[44] Zottler E.-M., Bieri M., Basso W., Schnyder M. (2019). Intestinal Parasites and Lungworms in Stray, Shelter and Privately Owned Cats of Switzerland. Parasitol. Int..

[45] Morelli S., Diakou A., Colombo M., Di Cesare A., Barlaam A., Dimzas D., Traversa D. (2021). Cat Respiratory Nematodes: Current Knowledge, Novel Data and Warranted Studies on Clinical Features, Treatment and Control. Pathogens.

[46] Pennisi M.G., Hartmann K., Addie D.D., Boucraut-Baralon C., Egberink H., Frymus T., Gruffydd-Jones T., Horzinek M.C., Hosie M.J., Lloret A. (2015). Lungworm Disease in Cats: ABCD Guidelines on Prevention and Management. J. Feline Med. Surg..

[47] Lélu M., Langlais M., Poulle M.-L., Gilot-Fromont E. (2010). Transmission Dynamics of *Toxoplasma gondii* along an Urban–Rural Gradient. Theor. Popul. Biol..

[48] Ruiz-Villar H., Bastianelli M.L., Heurich M., Anile S., Díaz-Ruiz F., Ferreras P., Götz M., Herrmann M., Jerosch S., Jubete F. (2023). Agriculture Intensity and Landscape Configuration Influence the Spatial Use of Wildcats across Europe. Biol. Conserv..

[49] McKenna P.B. (1999). Comparative Evaluation of Two Emigration/Sedimentation Techniques for the Recovery of Dictyocaulid and Protostrongylid Larvae from Faeces. Vet. Parasitol..

[50] R Core Team (2024). R: A Language and Environment for Statistical Computing.

[51] Gueldner E.K., Gilli U., Strube C., Schnyder M. (2019). Seroprevalence, Biogeographic Distribution and Risk Factors for *Aelurostrongylus abstrusus* Infections in Swiss Cats. Vet. Parasitol..

[52] Vanalli C., Mari L., Casagrandi R., Gatto M., Cattadori I.M. (2024). Helminth Ecological Requirements Shape the Impact of Climate Change on the Hazard of Infection. Ecol. Lett..

[53] Beugnet F., Bourdeau P., Chalvet-Monfray K., Cozma V., Farkas R., Guillot J., Halos L., Joachim A., Losson B., Miró G. (2014). Parasites of Domestic Owned Cats in Europe: Co-Infestations and Risk Factors. Parasit Vectors.

[54] Falsone L., Brianti E., Gaglio G., Napoli E., Anile S., Mallia E., Giannelli A., Poglayen G., Giannetto S., Otranto D. (2014). The European Wildcats (*Felis silvestris silvestris*) as Reservoir Hosts of *Troglostrongylus brevior* (Strongylida: Crenosomatidae) Lungworms. Vet. Parasitol..

[55] Veronesi F., Traversa D., Lepri E., Morganti G., Vercillo F., Grelli D., Cassini R., Marangi M., Iorio R., Ragni B. (2016). Occurrence of Lungworms in European Wildcats (*Felis silvestris silvestris*) of Central Italy. J. Wildl. Dis..

[56] Segeritz L., Anders O., Middelhoff T.L., Winterfeld D.T., Maksimov P., Schares G., Conraths F.J., Taubert A., Hermosilla C. (2021). New Insights into Gastrointestinal and Pulmonary Parasitofauna of Wild Eurasian Lynx (*Lynx lynx*) in the Harz Mountains of Germany. Pathogens.

[57] Traversa D., Morelli S., Di Cesare A., Diakou A. (2021). Felid Cardiopulmonary Nematodes: Dilemmas Solved and New Questions Posed. Pathogens.

[58] Gerngross P., Ambarli H., Angelici F.M., Anile S., Campbell R., Ferreras de Andres P., Gil-Sanchez J.M., Götz M., Jerosch M., Mengüllüoglu D. *Felis silvestris*. The IUCN Red List of Threatened Species 2023. https://www.iucnredlist.org.

[59] Barroso P., Relimpio D., Zearra J.A., Cerón J.J., Palencia P., Cardoso B., Ferreras E., Escobar M., Cáceres G., López-Olvera J.R. (2023). Using Integrated Wildlife Monitoring to Prevent Future Pandemics through One Health Approach. One Health.

[60] Traversa D., Di Cesare A. (2013). Feline Lungworms: What a Dilemma. Trends Parasitol..

[61] Hansen A.P., Skarbye L.K., Vinther L.M., Willesen J.L., Pipper C.B., Olsen C.S., Mejer H. (2017). Occurrence and Clinical Significance of *Aelurostrongylus abstrusus* and Other Endoparasites in Danish Cats. Vet. Parasitol..

[62] Lepczyk C.A., Fantle-Lepczyk J.E., Dunham K.D., Bonnaud E., Lindner J., Doherty T.S., Woinarski J.C.Z. (2023). A Global Synthesis and Assessment of Free-Ranging Domestic Cat Diet. Nat. Commun..

[63] Loss S.R., Will T., Marra P.P. (2013). The Impact of Free-Ranging Domestic Cats on Wildlife of the United States. Nat. Commun..

[64] Meli M.L., Cattori V., Martínez F., López G., Vargas A., Palomares F., López-Bao J.V., Hofmann-Lehmann R., Lutz H. (2010). Feline Leukemia Virus Infection: A Threat for the Survival of the Critically Endangered Iberian Lynx (*Lynx pardinus*). Vet. Immunol. Immunopathol..

[65] VanWormer E., Conrad P.A., Miller M.A., Melli A.C., Carpenter T.E., Mazet J.A.K. (2013). *Toxoplasma gondii*, Source to Sea: Higher Contribution of Domestic Felids to Terrestrial Parasite Loading Despite Lower Infection Prevalence. EcoHealth.

[66] Flockhart D.T.T., Coe J.B. (2018). Multistate Matrix Population Model to Assess the Contributions and Impacts on Population Abundance of Domestic Cats in Urban Areas Including Owned Cats, Unowned Cats, and Cats in Shelters. PLoS ONE.

[67] Soto I., Balzani P., Oficialdegui F.J., Molinero C., Kouba A., Ahmed D.A., Turbelin A.J., Hudgins E.J., Bodey T.W., Gojery S.A. (2024). The Wild Cost of Invasive Feral Animals Worldwide. Sci. Total Environ..

[68] Trouwborst A., McCormack P.C., Martínez Camacho E. (2020). Domestic Cats and Their Impacts on Biodiversity: A Blind Spot in the Application of Nature Conservation Law. People Nat..

[69] (2023). BOE Ley 7/2023, de 28 de Marzo, de Protección de los Derechos y el Bienestar de los Animales. https://www.boe.es/eli/es/l/2023/03/28/7/con.

[70] Hostetler M., Wisely S.M., Johnson S., Pienaar E., Main M. (2020). How Effective and Humane Is Trap-Neuter-Release (TNR) for Feral Cats?. EDIS.

[71] De Almeida Carvalho E.A., Rocha R.L. (2014). Visceral Larva Migrans Syndromes Associated with Toxocariasis: Epidemiology, Clinical and Laboratory Aspects of Human Toxocariasis. Curr. Trop. Med. Rep..

[72] Genchi M., Ferrari N., Fonti P., De Francesco I., Piazza C., Viglietti A. (2014). Relation between *Aelurostrongylus abstrusus* Larvae Excretion, Respiratory and Radiographic Signs in Naturally Infected Cats. Vet. Parasitol..

